# Analysis of the acoustoelectric response of SAW gas sensors using a COM model

**DOI:** 10.1038/s41378-024-00673-w

**Published:** 2024-05-24

**Authors:** Yang Yuan, Tao Yang, Xi Chen, Linglang Yu, Xiaoxiao Hou, Guangzu Zhang, Wen Dong, Zixiao Lu, Honglang Li, Leonhard Reindl, Wei Luo

**Affiliations:** 1 School of Integrated Circuits, Huazhong University of Science and Technology, 430074 Wuhan, People’s Republic of China; 2 CAS Center for Excellence in Nanoscience, National Center for Nanoscience and Technology, 100190 Beijing, People’s Republic of China; 3 Department of Microsystems Engineering, Laboratory for Electrical Instrumentation and Embedded Systems, University of Freiburg, 79110 Freiburg, Germany; 4 Research Institute of Huazhong University of Science and Technology in Shenzhen, 518057 Shenzhen, People’s Republic of China

**Keywords:** Sensors, Electrical and electronic engineering

## Abstract

Surface acoustic wave (SAW) gas sensors based on the acoustoelectric effect exhibit wide application prospects for in situ gas detection. However, establishing accurate models for calculating the scattering parameters of SAW gas sensors remains a challenge. Here, we present a coupling of modes (COM) model that includes the acoustoelectric effect and specifically explains the nonmonotonic variation in the center frequency with respect to the sensing film’s sheet conductivity. Several sensing parameters of the gas sensors, including the center frequency, insertion loss, and phase, were experimentally compared for accuracy and practicality. Finally, the frequency of the phase extremum (FPE) shift was determined to vary monotonically, and the range of selectable test points was wide, making the FPE an appropriate response parameter for leveraging in SAW gas sensors. The simulation results of the COM model were highly consistent with the experimental results. Our study is proposed to provide theoretical guidance for the future development of gas SAW sensors.

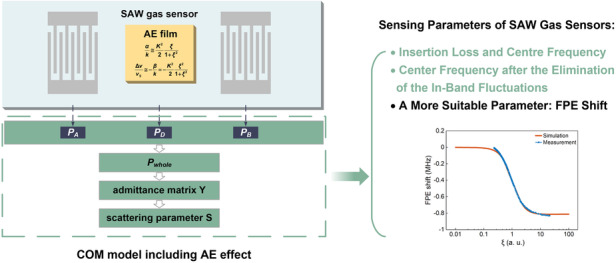

## Introduction

Recently, surface acoustic wave (SAW) gas sensors have attracted significant attention across numerous industries due to their advantages, such as high sensitivity, digital output, reliability, accuracy, low cost, and passive wireless detection^1,2^. SAW gas sensors typically incorporate one or two of the following sensing mechanisms: the mass loading effect, the elastic loading effect, or the acoustoelectric effect^3–5^. Most studies have utilized frequency shift equations to analyze the dependence of mass, elastic modulus, and conductivity on the frequency shift of SAW gas sensors toward determining the primary underlying mechanisms of these techniques^6,7^. Recent studies have indicated that the acoustoelectric effect plays a more significant role in influencing the frequency shift as depends on the acoustic velocity^8^. Therefore, several studies have attempted to use theoretical analysis to optimize the performance of SAW sensors based on the acoustoelectric effect. Most of these related studies have relied solely on velocity change equations to calculate the center frequency shift of SAW sensors^9,10^, while some have attempted to establish corresponding models. Jakubik et al. developed a phenomenological model outlining the interdependence between normalized sheet conductivity and acoustoelectric sensitivity^11^. The model predicted the performance of SAW gas sensors with single or bilayer structures. Using the transfer matrix method, Fan et al. calculated the effect of sheet conductivity on the velocity and center frequency of a multilayer SAW sensor^12^. However, these studies did not consider the center frequency shifts caused by triple transit signals, electrode reflections, or other stray signals.

While the abovementioned theoretical analyses have contributed to improving the sensitivity of sensors, they have limitations and cannot fully explain some experimental phenomena for SAW gas sensors based on the acoustoelectric effect. For example, while some experimental results are consistent with the theoretical predictions that the center frequency of SAW sensors decreases as the film sheet conductivity increases^13,14^, other studies reported conflicting results on this topic^15,16^. Thus, it is inaccurate to predict frequency shifts solely based on the velocity changes caused by the acoustoelectric effect in SAW sensors. The center frequency^17–19^ is the most commonly used sensing parameter for SAW gas sensors, and some studies have investigated the variations in insertion loss^20^ and phase^21,22^ induced by target gases. However, existing models cannot simultaneously calculate these sensing parameters. Based on the aforementioned analysis, a major issue in SAW sensor modeling is the absence of a solution for calculating the transmission coefficient of SAW sensors while accounting for the influence of the film sheet conductivity.

In this work, we establish a model that includes the acoustoelectric effect for SAW gas sensors. The model incorporates film sheet conductivity changes into the *P*-matrix of the acoustic propagation path, enabling the calculation of the scattering parameters for the entire sensor system. The model also couples second-order effects such as electrode reflections, wave velocity variations, and propagation losses. Compared to the finite element method (FEM), which is known for its high precision in SAW device analysis, the coupling of modes (COM) model provides an efficient alternative for simulating the transmission coefficient without imposing significant computational and storage burdens. The modified COM model can simulate the variations in the sensing parameters of SAW gas sensors, including the center frequency, insertion loss, and phase. The simulation and experimental results exhibit excellent agreement, confirming the accuracy of the proposed model. This model was used to simulate the nonmonotonic variation between the center frequency and sheet conductivity, providing theoretical support for previously unexplained experimental phenomena. Additionally, the step changes in the center frequency induced by in-band fluctuations are investigated. Furthermore, the FPE shift serves as a suitable response parameter for SAW gas sensors due to its monotonic variation within a wide range of thin-film conductivities.

## Model description

### Acoustoelectric effect

The interaction between SAWs and carriers in an overlaid semiconductor can be described using a complex propagation parameter as *γ* = *α* + *jβ*. The real part *α* and the imaginary part *β*, each of which are nonmonotonic functions of the normalized sheet conductivity *ξ*, can be used to determine the attenuation per wavenumber *k* and the fractional velocity perturbation as follows^23^:

1 $$\frac{\alpha }{k}\cong \frac{{K}^{2}}{2}\frac{\xi }{1+{\xi }^{2}}$$

2 $$\frac{\varDelta v}{{v}_{S}}\cong -\frac{\beta }{k}=-\frac{{K}^{2}}{2}\frac{{\xi }^{2}}{1+{\xi }^{2}}$$

where *K*^2^ is the electromechanical coupling coefficient, $\xi ={\sigma }_{S}/{\sigma }_{M}$; *σ*_*s*_ is the sheet conductivity of the sensing film; *σ*_*M*_ is the characteristic sheet conductivity at which the maximum acoustoelectric attenuation and the greatest rate of change in acoustic velocity both occur; *σ*_*M*_ is given by ${\sigma }_{M}={v}_{S}({\varepsilon }_{0}+\varepsilon )$, where *v*_*S*_ is the acoustic velocity, *ε*_0_ is the vacuum dielectric constant, *ε* is the dielectric constant of the piezoelectric medium, and Δ*v* is the change in acoustic velocity due to exposure of the sensing film to the target gases. Based on Eqs. (1) and (2), Fig. S1 was plotted to depict the amplitude attenuation and velocity shift of the SAW variation as a function of *ξ*. The acoustoelectric effect becomes more pronounced with increasing *K*^2^. When *ξ* = 1, the film carriers gain the most energy, which results in the most attenuation and the fastest velocity change.

An oscillating electric field excited by the acoustic surface wave propagates across the piezoelectric substrate and interacts with carriers in the semiconductor layer, resulting in coupling between the phonons and electrons. The film sheet conductance varies due to the target gas, changing the transmission coefficient S_21_ response of the sensor.

### Establishment of the COM model for SAW gas sensors

**Fig. 1:**
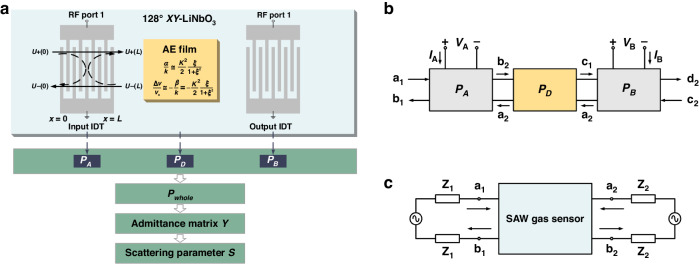
Schematic of the simulation studies on the SAW delay line gas sensor. **a** Schematic of the geometric layout and associated COM model. **b** P-matrix cascading. **c** Equivalent two-terminal network for a SAW gas sensor

The COM model was established to calculate the S_21_ response of the SAW delay line gas sensors, as shown in Fig. 1a. The input interdigital transducer (IDT), acoustic propagation path and output IDT are the three fundamental components of gas sensors. Building upon the existing COM model for the SAW delay line, this study expanded the existing model to incorporate the impact of thin films on the energy and velocity of SAWs within the acoustic transmission path. The corresponding investigation focused on analyzing the influence of the acoustoelectric effect on the gas sensor response, encompassing parameters such as insertion loss, center frequency, and phase.

The process of excitation, transmission, and interaction of SAWs in an IDT can be described by the *P*-matrix. Based on the external operating characteristics, the IDT is equated to a three-port linear circuit comprising an electrical terminal and two acoustic terminals. The *P*-matrix of the IDT is expressed as follows:

3 $$\left[\begin{array}{c}{{\rm{U}}}_{-}\left(0\right)\\ {{\rm{U}}}_{+}\left({\rm{L}}\right)\\ {\rm{I}}\end{array}\right]=\left[\begin{array}{ccc}{{\rm{P}}}_{11} & {{\rm{P}}}_{12} & {{\rm{P}}}_{13}\\ {{\rm{P}}}_{21} & {{\rm{P}}}_{22} & {{\rm{P}}}_{23}\\ {{\rm{P}}}_{31} & {{\rm{P}}}_{32} & {{\rm{P}}}_{33}\end{array}\right]\left[\begin{array}{c}{{\rm{U}}}_{+}\left(0\right)\\ {{\rm{U}}}_{-}\left({\rm{L}}\right)\\ {\rm{V}}\end{array}\right]$$

where *U*_+_ (*x*) and *U*_(*x*) are the forward and backward propagation amplitudes of the SAW, *L* is the length of the transducer, *I* is the current, and *V* is the voltage.

The basic equations of the COM model include a group of partial differential equations that describe the coupling of SAWs propagating in opposite directions. The solution of the COM equation for the IDT can be expressed in terms of the *P*-matrix, and each element in the matrix can be represented by COM parameters^24^. The COM parameters that depend mainly on the piezoelectric substrate are obtained from Abbott and Hashimoto’s frequency dispersion equations and Plessky’s dispersion equations for a two-parameter model^25,26^.

In addition, the *P*-matrix of the acoustic propagation path for the SAW delay line is expressed as follows:

4 $${P}_{D}=\left[\begin{array}{ccc}0 & D & 0\\ D & 0 & 0\\ 0 & 0 & 0\end{array}\right]$$

For a free surface, ${D=e}^{-j{k}_{f}{L}_{d}}$, where *k*_*f*_ is the free surface propagation constant and *L*_*d*_ is the propagation path length. *k*_*f*_ is given by ${k}_{f}=\frac{\omega }{{v}_{s}}-j{{\rm{\gamma }}}^{{\prime} }$, where *ω* is the angular frequency, *v*_*s*_ is the acoustic velocity, and *γ*′ is the propagation loss. If the acoustic propagation path is covered by a semiconductor film, it is necessary to consider the attenuation and the change in SAW velocity that results from the acoustoelectric effect, which is represented by the propagation parameter *γ* = *α* + *jβ*. The corresponding modified expression is given by $D={e}^{-j{k}_{f}{L}_{d}-\gamma L}={e}^{-j\left(\frac{\omega }{{v}_{s}}-j{{\rm{\gamma }}}^{{\prime} }\right){L}_{d}-\left(\frac{\xi }{1+{\xi }^{2}}+j\frac{{\xi }^{2}}{1+{\xi }^{2}}\right)k\frac{{K}^{2}}{2}L}$, where *L* is the length of the semiconductor film along the direction of acoustic propagation.

The acoustic propagation path has no electrical terminal, and its acoustic terminals are connected in series with the acoustic terminals of the input and output IDTs, as shown in Fig. 1b. The *P*-matrix of the SAW delay line gas sensors is derived from the unit *P*-matrix by cascading. The admittance matrix *Y* and scattering parameters *S* of the gas sensors are obtained from the *P*-matrix of the SAW delay line^27^. The admittance matrix *Y* is expressed as follows:

5 $$\left[\begin{array}{c}{I}_{A}\\ {I}_{B}\end{array}\right]=\left[\begin{array}{cc}{Y}_{11} & {Y}_{12}\\ {Y}_{21} & {Y}_{22}\end{array}\right]\left[\begin{array}{c}{V}_{A}\\ {V}_{B}\end{array}\right]$$

where *V*_*A*_ and *V*_*B*_ are the voltages applied to the electrical terminals of the input and output IDTs, respectively, and *I*_*A*_ and *I*_*B*_ are the currents generated by the input and output IDTs, respectively.

The relationship between the admittance parameter *Y*_*ij*_ and the individual elements *P*_*ij*_ of the *P*-matrices is expressed as follows:

6 $${Y}_{11}={P}_{33}^{A}+\frac{{P}_{23}^{A}{P}_{32}^{A}{P}_{11}^{B}{D}^{2}}{1-{P}_{22}^{A}{P}_{11}^{B}{D}^{2}}$$

7 $${Y}_{22}={P}_{33}^{B}+\frac{{P}_{13}^{B}{P}_{31}^{B}{P}_{22}^{A}{D}^{2}}{1-{P}_{22}^{A}{P}_{11}^{B}{D}^{2}}$$

8 $${Y}_{12}{=Y}_{21}=\frac{{P}_{13}^{B}{P}_{32}^{A}D}{1-{P}_{22}^{A}{P}_{11}^{B}{D}^{2}}$$

Finally, the SAW gas sensor can be regarded as part of a two-terminal network, as shown in Fig. 1c. Based on the admittance matrix *Y*, the transmission coefficient *S*_21_, which includes both magnitude and phase, is expressed as follows:

9 $${S}_{21}=\frac{-2\sqrt{{R}_{1}{R}_{2}}{Y}_{21}}{\left(1+{Z}_{1}{Y}_{11}\right)\left(1+{Z}_{2}{Y}_{22}\right)-{Z}_{1}{Z}_{2}{{Y}_{12}Y}_{21}}$$

where ${R}_{i}=\mathrm{Re}\left({Z}_{i}\right)$ and Z_*i*_ is the equivalent impedance of the peripheral circuits.

According to the COM model for SAW gas sensors, *ξ* influences element *D* of the *P*_*D*_ matrix, thereby influencing the admittance matrix *Y* and the scattering parameter *S*_21_.

## Experimental section

### SAW sensor fabrication

**Fig. 2:**
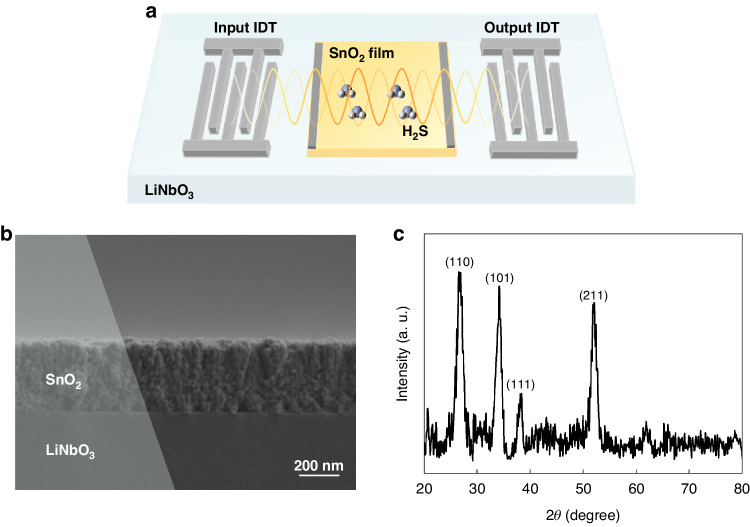
SAW gas sensor overview. **a** Schematic of the acoustoelectric effect for a thin film on an LN substrate. **b** SEM image of the SnO_2_/LN sample. **c** XRD pattern of the SnO_2_/LN sample

Based on the simulation results of the COM model, a prototype SAW gas sensor was fabricated. Figure 2a schematically depicts the structure of the acoustoelectric effect for a SnO_2_ thin film on a 128° YX-LiNbO_3_ (LN) substrate. The IDT design was patterned by photolithography. Each IDT comprised 30 finger pairs with a period of 20 µm. A Cr/Al (5 nm/600 nm) bilayer was subsequently deposited on the wafer using an e-beam evaporator, followed by a lift-off process to form the IDTs. The aperture of the IDTs was 500 μm, and the propagation length was 4000 μm. The main sensing mechanism of SAW H_2_S sensors based on SnO_2_ films is the acoustoelectric effect^7^. This sensor serves as a representative example to validate the wide applicability and reliability of the modified model. To realize the sensing film, a 0.7 × 2 mm^2^ SnO_2_ film was deposited in the acoustic propagation region of the SAW sensor. Ag electrodes with a thickness of 200 nm for testing the film resistance were deposited on both sides of the SnO_2_ film using vacuum evaporation.

### Material deposition

The SnO_2_ film was deposited on an LN substrate at room temperature by RF magnetron sputtering of the SnO_2_ target (diameter: 3 in, purity: 99.99%). The distance between the target and substrate was 100 mm. The base pressure for deposition was less than 9 × 10^−4^ Pa, and during deposition, and this pressure was maintained at 2 Pa using a 30 sccm Ar constant flow and an adaptive pressure controller. The SnO_2_ target was set at 100 W. The SnO_2_ film deposition rate was 7 nm/min. Finally, the samples were annealed at 500 °C for 2 h. The prepared SnO_2_ film exhibited a strong response to H_2_S, which manifested as a wide range of sheet conductivity changes and realized a SnO_2_ film that was suitable for verifying the correctness of the COM model.

### Characterization

Scanning electron microscopy (SEM, ZEISS, GeminiSEM 300) was used to inspect the SnO_2_ layer morphologies. As shown in Fig. 2b, the SnO_2_ layer exhibited a homogeneous and dense microstructure with a uniform thickness of 350 nm. The crystal structure of the SnO_2_ layer was analyzed via X-ray diffraction (XRD, Empyrean, PANalytical B.V.) with Cu Kα radiation in the 2*θ* range of 10–80°. As shown in Fig. 2c, four distinct 2*θ* peaks at 26.61°, 33.89°, 38.97°, and 51.78° were attributed to the (110), (101), (111), and (211) planes, respectively. According to Hall measurements (ECOPIA HMS-5500), the SnO_2_ layer exhibited an electron mobility of 1.85 cm^2^/(V·s), a sheet carrier density *n*_*s*_ of 1.58 × 10^12 ^cm^−2^, and a sheet conductance of 4.68 × 10^−7^ S at RT.

### Experimental setup

**Fig. 3:**
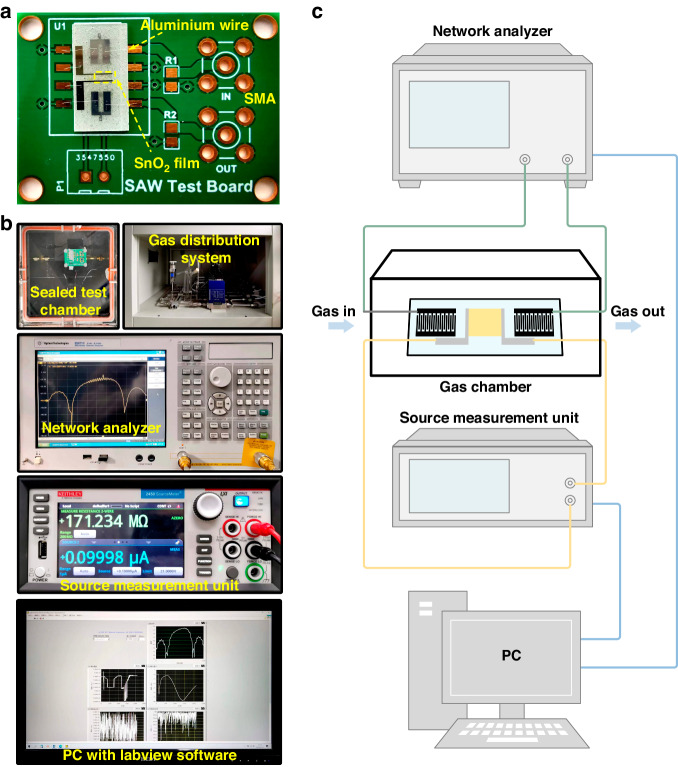
SAW gas sensing test system. **a** Specially designed PCB for SAW sensor measurements. **b** Image and **c** schematic illustration of the gas sensing test system

In Fig. 3a, the SAW sensor was fixed on a specially designed printed circuit board. The gas sensing measurement system as described in Fig. 3b, c was used to evaluate the SAW gas sensor. This system was capable of dynamically regulating humidity and target gas concentration. The temperature and relative humidity in the test were maintained at 25 °C and 25%, respectively. During the experiments, the SnO_2_ film was exposed to 50 ppm H_2_S gas, and the total flow rate was adjusted to 200 sccm. The S_21_ of the SAW gas sensor under static conditions was determined via a network analyzer (Agilent E5071C). The film resistance was measured using a source measurement unit (Keithley 6487) while simultaneously measuring S_21_. The output signal was monitored in real-time using software. The SnO_2_ sheet conductivity was determined from its resistance *R*_*S*_ using the equation ${\sigma }_{S}=(1/{R}_{S})\times (W/L)$, where *W* and *L* are the width (0.7 mm) and length (2 mm) of the film, respectively.

## Results and discussion

### COM model validation

**Fig. 4:**
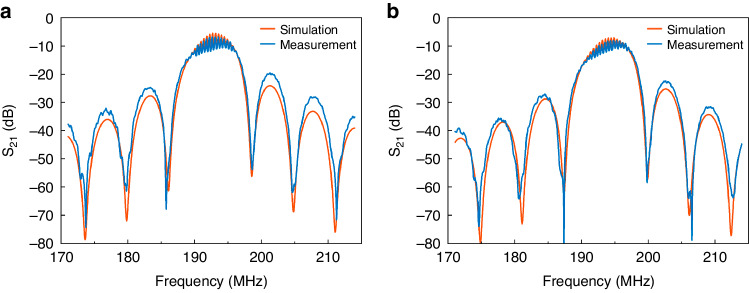
Simulated and measured S_21_. **a** Original SAW sensor and **b** the sensor coated with the SnO_2_ film

The simulated and measured S_21_ curves of the SAW gas sensor before and after depositing the SnO_2_ thin film are shown in Fig. 4. Details of the LN parameters used in the COM model are listed in Table 1. We determined the insertion loss and center frequency based on the S_21_ curve, where the center frequency was defined as the frequency corresponding to the minimum insertion loss. The S_21_ curves obtained from both the measurements and simulations exhibited similar results. Reflections at the fingered electrodes led to asymmetry in the main lobe of the S_21_ curves. The measured insertion losses were slightly greater than the simulated insertion losses due to intrinsic limitations of the physical device, such as nonideal energy losses and incomplete impedance matching due to the effects of the measurement system. The simulated and measured scattering properties of the designed SAW gas sensor with and without the SnO_2_ thin film are summarized in Table 2. The measured center frequencies of the device without and with the SnO_2_ thin film were 193.06 MHz and 194.81 MHz, respectively. When the SAW coupled to the SnO_2_ thin film, part of the SAW acoustic energy was transferred into the film. The measured insertion loss increased from 6.77 dB to 8.05 dB, which is attributed to the acoustoelectric effect. There was no significant difference between the simulated and measured results, verifying the accuracy of the modified COM model. The above results demonstrated that the COM model can accurately simulate the frequency response characteristics of the prototype SAW gas sensor, establishing the model as a tool for optimizing sensor structures.

**Table 1:** The 128° YX-LiNbO_3_ parameters used in the COM simulation

Parameters	Values
Acoustic velocity *v*_*S*_ (m/s)	3992
Electromechanical coupling coefficient *K*^2^ (%)	5.5
Dielectric constant *ε* = 56.68*ε*_0_ (F/m)	5.02 × 10^−10^
Characteristic sheet conductivity *σ*_*M*_ (S)	2.04 × 10^−6^

**Table 2:** The simulation and measurement results of the center frequency and insertion loss without and with the sensing film are shown in Fig. 4

Structures	Methods	Center frequency (MHz)	Insertion loss (dB)
Without film	Simulation	192.70	−5.46
Without film	Measurement	193.06	−6.77
With film	Simulation	194.82	−7.04
With film	Measurement	194.81	−8.05

### Optimization of the SAW gas sensors

The modified COM model was further utilized to study three key aspects of SAW sensors. First, the variations in the insertion loss and center frequency with film sheet conductivity were examined. Second, the center frequency was determined to be susceptible to step changes caused by in-band fluctuations, and a time-gated technique was implemented to mitigate this effect. Finally, the phase frequency shift displayed a monotonic relationship with the film sheet conductivity, indicating that the phase frequency shift is a more favorable property to employ as a sensing parameter.

#### Insertion loss and center frequency

**Fig. 5:**
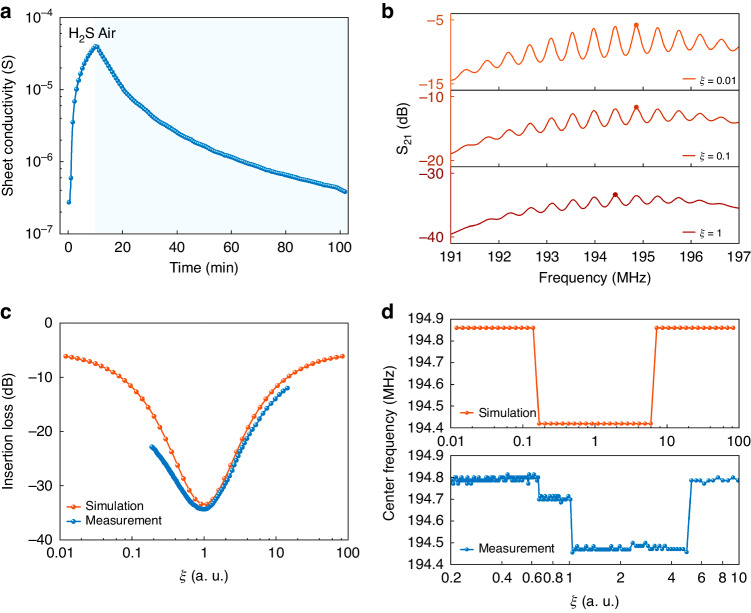
Frequency response characteristics. **a** Sensing curves of the film sheet conductivity. **b** Simulated S_21_ for *ξ* = 0.01, 0.1, and 1, where the center frequencies are marked. The simulated and measured **c** insertion loss and **d** center frequency as a function of *ξ*

The operating principle of SAW gas sensors based on the acoustoelectric effect relies on the film sheet conductivity changing due to the adsorption and desorption of target gas molecules, which affects the acoustic transmission characteristics. In the modified COM model, the normalized sheet conductivity *ξ* was used as the independent variable to calculate S_21_ and obtain the corresponding center frequency and insertion loss. A monotonically varying characteristic is typically needed as a measurement parameter for gas sensors. However, our simulations and experiments showed that the center frequency and loss varied nonmonotonically with *ξ*. The SnO_2_ sheet conductivity response and recovery curves of the SAW gas sensor toward H_2_S at room temperature are shown in Fig. 5a. The corresponding dynamic insertion loss and center frequency response curves of gas exposure and release are shown in Fig. S2. Due to the rapid response of the sensor in the experiment and the limited density of data points used for analysis, the recovery curve of the sensor was utilized for comparison with the simulation results. Based on these recovery curves, we obtained the insertion loss and center frequency as functions of *ξ* and compared these values with the simulated results, as shown in Fig. 5c, d. The insertion loss reached its maximum at *ξ* = 1 (*σ*_*S*_ = *σ*_*M*_). The simulated center frequency exhibited the following characteristics: (1) the trend of the curve was not monotonic but was rather symmetrical about *ξ* = 1, and (2) the curve was not smooth and had stepped shapes. Notably, the variations in film sheet conductivity and gas concentration are correlated, and within a certain concentration range, this relationship may exhibit linearity. As the concentration of H_2_S decreases, the range of variation in *ξ* will narrow. This phenomenon is further discussed by investigating the variations in the insertion loss at different H_2_S concentrations, as shown in Fig. S3. When the H_2_S concentration drops to a certain value, *ξ* remains consistently less than 1, and the variation in the insertion loss with *ξ* becomes monotonic.

Figure 5b shows the simulated S_21_ with significant in-band fluctuations based on the different film sheet conductivities. The in-band fluctuations in the S_21_ curves are caused by the combination of the direct signal with parasitic responses that propagate through the delay line multiple times with decreasing amplitude^28^. The exact position of these ripples depends on the phase relationship between the direct signal and multiple transit signals, which can be affected by changes in the SAW velocity (which may occur due to factors such as film sheet conductivity, electrical or mechanical boundary conditions, and temperature). As the film sheet conductivity changes, the in-band fluctuations are affected. The center frequency remains constant until there is a shifting of the wave peak where the minimum insertion loss occurs. This shift does not affect the continuous change in the insertion loss because it is approximately the same for several adjacent wave peaks. The insertion loss increases when *ξ* < 1 and the sheet conductivity increases. When the highest peak of the in-band fluctuation changes, this change results in a sudden shift of the center frequency. However, the experimentally observed step change in the central frequency is more pronounced than that observed in the simulation results, as shown in Fig. 5d. This discrepancy is attributed to the assumption of ideal conditions by the model, while second-order effects and parasitic responses of the device in the actual test can result in more significant changes in the in-band fluctuations. In gas sensing experiments, it is typical that the center frequency undergoes continuous variation when the sheet conductivity changes only to a limited extent and when the in-band fluctuation is negligible. In addition, if the response time of the SAW sensor is slow, step changes in the center frequency are more pronounced^29^.

#### Center frequency after elimination of in-band fluctuations

**Fig. 6:**
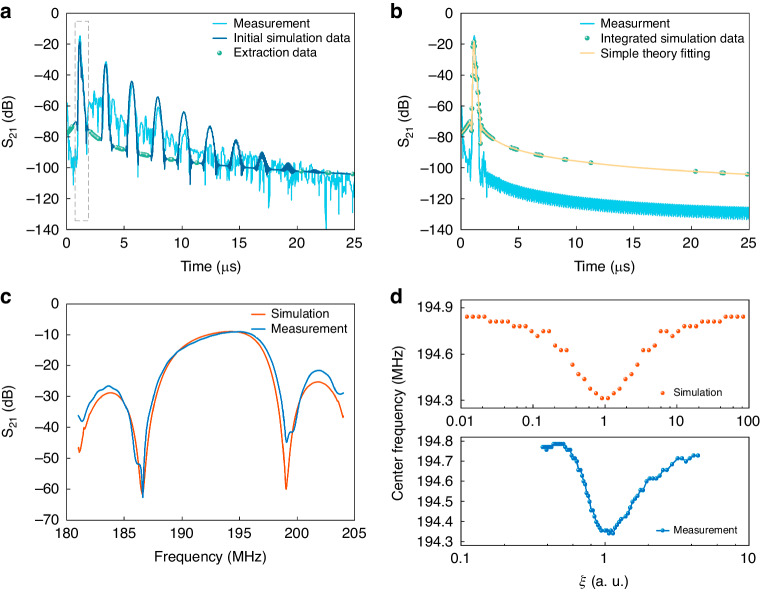
Simulation and measurement results of signal processing for the SAW gas sensor. In the case of no gas infusion, the S_21_ time domain response **a** before and **b** after processing using the time-gated technique of the Fourier transform operation and **c** the S_21_ frequency response after signal processing. **d** Center frequency after elimination of in-band fluctuations as a function of *ξ*

To investigate the physical mechanism underlying the center frequency step changes in Fig. 5d, the frequency domain S_21_ curve was filtered by the time-gating technique based on Fourier transform operations. The frequency domain S_21_ curve recorded for the case of no gas infusion (as obtained from the modified COM model shown in Fig. 4b) was converted to the time domain using a fast Fourier inverse transform, thereby deriving the pulse response of the sensor. As shown in Fig. 6a, the first pulse was the direct signal, and the subsequent continuous pulses were parasitic responses, due to effects such as the occurrence of multiple reflection signals and round-trip reflection signals. On a linear scale, the direct signal can be represented as a triangle with a slightly slower falling edge slope than a rising edge slope due to the reflections within the IDTs. The direct signal contains all the necessary information for evaluation, while any signals outside this region are considered interfering signals. Subsequently, the direct signal data and other data in the time domain response excluding the pulses were fit to obtain a new curve, as shown in Fig. 6b. Finally, based on the fitting curve and its phase information, a fast Fourier transform was performed to obtain the gated S_21_ signal in the frequency domain, as shown in Fig. 6c. In this experiment, the time-gated function of the network analyzer was utilized to eliminate unnecessary signals, and the processed frequency domain S_21_ curve did not exhibit in-band fluctuations, as were observed in the simulation results. The center frequency of the elimination of in-band fluctuations varied with *ξ*, as shown in Fig. 6d, with no step changes in the center frequency. The corresponding dynamic response curves of gas exposure and release are shown in Fig. S4. The above analysis revealed that multiple reflection signals and round-trip reflection signals were the main causes of in-band fluctuations, which ultimately led to step changes in the center frequency. As such, in-band fluctuations could be eliminated through signal processing or by optimizing the IDT structure, such as through the use of a single-phase unidirectional transducer (SPUDT). However, the dynamic curves of the center frequency after the elimination of in-band fluctuations in the simulation and experiment were not smooth. This occurs because even after the elimination of triple transit and any other interfering signals, the exact location of the minimum insertion loss remains somewhat affected by the internal reflections within the IDT. Theoretical predictions of the center frequency shift, as shown in Fig. 6d, exhibited a nonmonotonic trend that was consistent with the experimental results. Specifically, as *ξ* increases, the center frequency initially decreases ($\xi < 1$, ${\sigma }_{S} < {\sigma }_{M}$) and then increases ($\xi > 1$, ${\sigma }_{S} > {\sigma }_{M}$) and is determined by the relative magnitudes of *σ*_*S*_ and *σ*_*M*_. In Eq. (2), the velocity change is inversely proportional to *ξ*. Moreover, as the center frequency shift is directly proportional to the velocity change, the center frequency shift is inversely proportional to the normalized sheet conductivity. Therefore, Eq. (2) could not exclusively explain why the observed phenomenon of the sheet conductance was proportional to the frequency in this work or in Refs. ^15,16^, which were caused by the acoustoelectric effect. In contrast, the modified COM model directly simulated the center frequency shift, overcoming the limitations of the equation-based description and providing a more accurate explanation of the frequency response of the SAW gas sensor.

#### A more suitable parameter: frequency of phase extremum shift

**Fig. 7:**
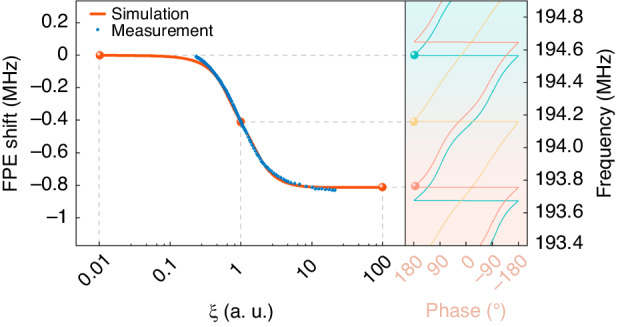
Simulated and measured FPE shift as a function of *ξ*. The FPE was simulated with *ξ* = 0.01, 0.1, and 1, and the change points were marked accordingly

The insertion loss and center frequency are commonly used as sensing parameters in SAW wave gas sensors. If the resistance of the sensing film changes significantly in the case of gas infusion, the changes in the insertion loss and center frequency that occur after the elimination of in-band fluctuations are not monotonic. Therefore, the insertion loss and center frequency do not satisfy the requirements of the sensing parameters. In some previous studies, the phase shift is used as the sensing parameter for gas testing. However, due to the limited range of the phase shift (−180° to 180°), which leads to complex experimental data processing, we propose and discuss the possibility of using the FPE shift as a sensing parameter. In our experiment, a phase extremum near the center frequency was marked with a network analyzer, and the frequency shift of this marked point was recorded when the sensor was exposed to H_2_S. The corresponding dynamic response curves of gas exposure and release are shown in Fig. S5. The modified COM model was used to simulate FPE corresponding to different *ξ* values, and there was high consistency between the simulated and experimental results (as shown in Fig. 7), further demonstrating the accuracy of the modified COM model. As *ξ* increased, the FPE changed monotonically, and when *ξ* = 1, the FPE changed very quickly, indicating the highest sensitivity of the sensor.

In SAW gas sensors, the adsorption of gas induces a phase shift ΔΦ between two IDTs, stemming from the change in velocity of the SAW (*Δv*/*v*_*S*_). Specifically, this relationship is expressed as follows:

10 $$\Delta \Phi =2{\rm{\pi }}f\times \frac{l}{{v}_{S}}\times \frac{\varDelta v}{{v}_{S}}$$

where *f* is the frequency and *l* is the distance in the acoustic propagation region.

In Fig. 7, the phase angle is held constant at the marked point, and the resulting frequency shift is recorded. The angle and frequency are different representations of the same phase change and exhibit the same trend. Therefore, the FPE should demonstrate a similar trend as the variation in acoustic velocity. Crucially, the center frequency, which is typically defined as the frequency corresponding to the maximum point on the S_21_ curve, is influenced by changes in the SAW velocity and by SAW attenuation. Thus, even when eliminating in-band fluctuations, the trend of the center frequency shift differs from that of the velocity change.

The phase experiment was independent of signal processing, which resulted in more convenient and quicker gas detection than does the evaluation of the center frequency. Furthermore, the more substantial shift in the FPE, in contrast to the shift in the center frequency, significantly enhances the gas sensor response, leading to heightened sensitivity. As a sensing parameter, the FPE had no specific requirements for the range of resistance changes and showed good monotonicity. In addition, the selection of test points for the frequency of the phase shift was broad. As the film sheet conductivity increased, the phase curve shifted toward lower frequencies without significant changes in shape, as reflected in the simulated phase curves shown in Fig. 7. Therefore, any point on the phase curve measurable by the SAW sensor can be a viable test point for investigating the frequency associated with the phase shift, which broadened the selection range of testing.

## Conclusion

Our study established a COM model that included the acoustoelectric effect for SAW gas sensors. This model is proposed to serve as a valuable tool for optimizing sensor performance. The performance of the model was experimentally validated, and a comparison revealed that the measured and simulated results were strongly correlated. The modified COM model was also used to explain previously reported nonmonotonic variations in the center frequency for several SAW gas sensors based on the acoustoelectric effect. In the present state of the art, there is a lack of simulation models for calculating the phase of SAW gas sensors, and our study effectively addresses this issue. Additionally, the phase frequency shift was demonstrated to be a more suitable sensing parameter for use with SAW gas sensors.

### Supplementary information

Supplemental Material File #1
