# Supplementary material for: Analysis of the acoustoelectric response of SAW gas sensors using a COM model
Source: Microsyst Nanoeng. 2024 May 24;10:69. doi: 10.1038/s41378-024-00673-w (PMC11126560; doi:10.1038/s41378-024-00673-w)
Supplement: Supplementary file 1 — Supplemental Material File #1 [file 41378_2024_673_MOESM1_ESM.docx]

Analysis of the Acoustoelectric Response of SAW Gas Sensors Using a COM Model

Yang Yuan^1^, Tao Yang^1^, Xi Chen^1^, Linglang Yu^1^, Xiaoxiao Hou^1^, Guangzu Zhang^1^, Wen Dong^1^, Zixiao Lu^2^, Honglang Li^2^, Leonhard Reindl^3^, and Wei Luo^1,4,*^

^1^ School of Integrated Circuits, Huazhong University of Science and Technology, Wuhan 430074, PR China

^2^ CAS Center for Excellence in Nanoscience, National Center for Nanoscience and Technology, Beijing 100190, PR China

^3^ Department of Microsystems Engineering, Laboratory for Electrical Instrumentation and Embedded Systems, University of Freiburg, Freiburg 79110, Germany

^4^ Research Institute of Huazhong University of Science and Technology in Shenzhen, Shenzhen 518057, PR China

**Fig. S1.** (a) The amplitude attenuation and (b) velocity shift of the SAW variation as a function of 𝜉.


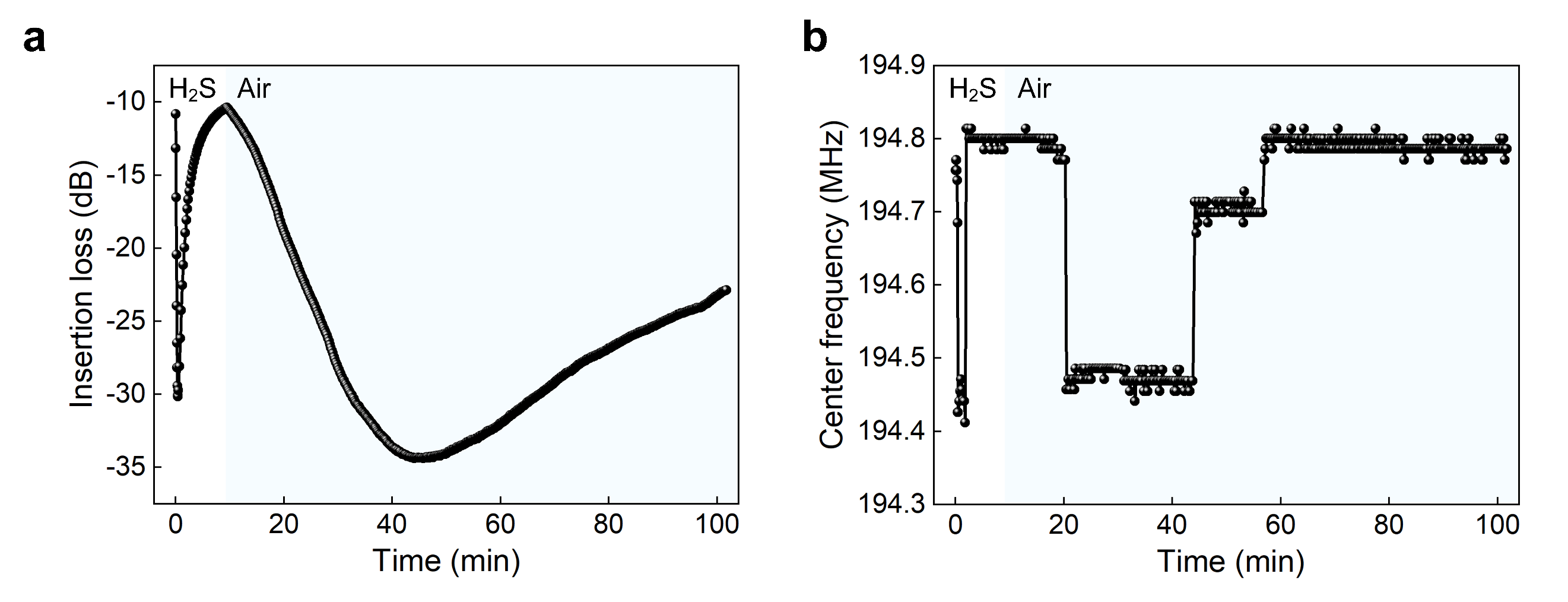


**Fig. S2.** The time domain sensing curves of the (a) insertion loss and (b) center frequency, correspond to Fig. 5.

**Fig. S3.** The simulated and measured insertion loss as a function of $\xi$, with H2S concentrations of 50, 20, and 2 ppm during testing.


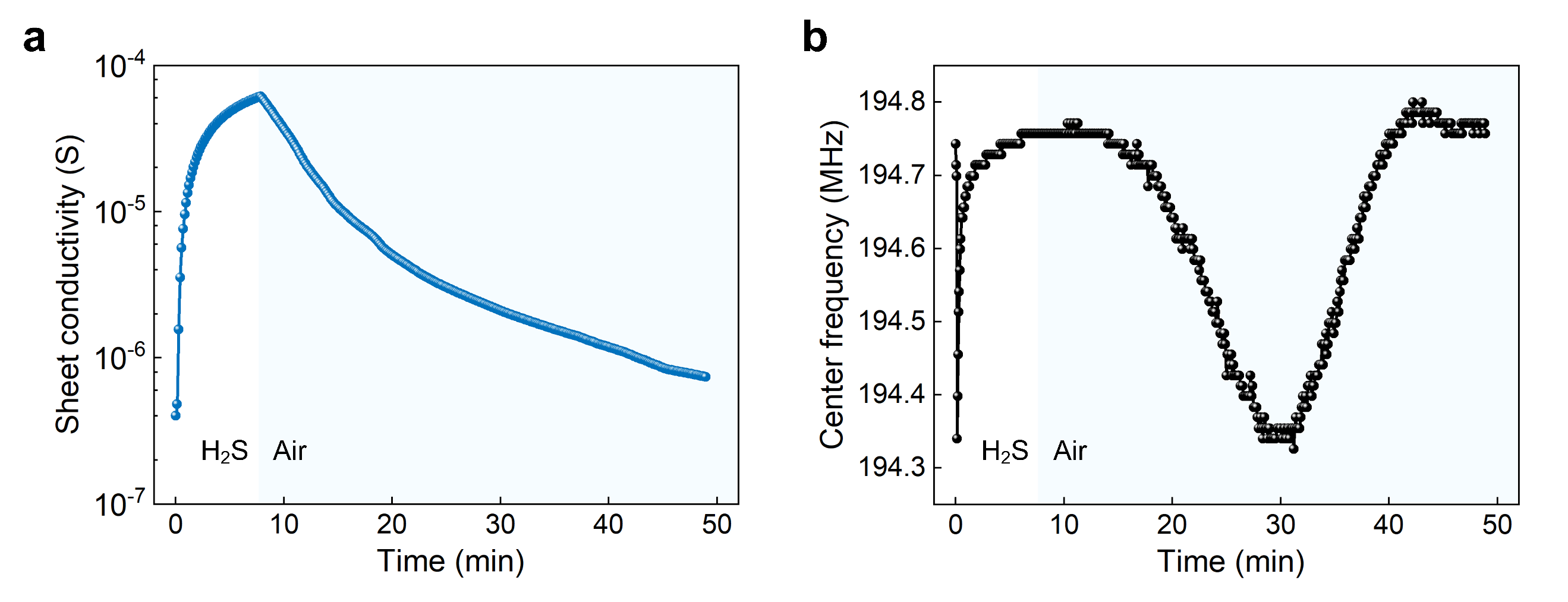


**Fig. S4.** The time domain sensing curves of the (a) film sheet conductivity and (b) center frequency after the elimination of in-band fluctuations, correspond to Fig. 5d.


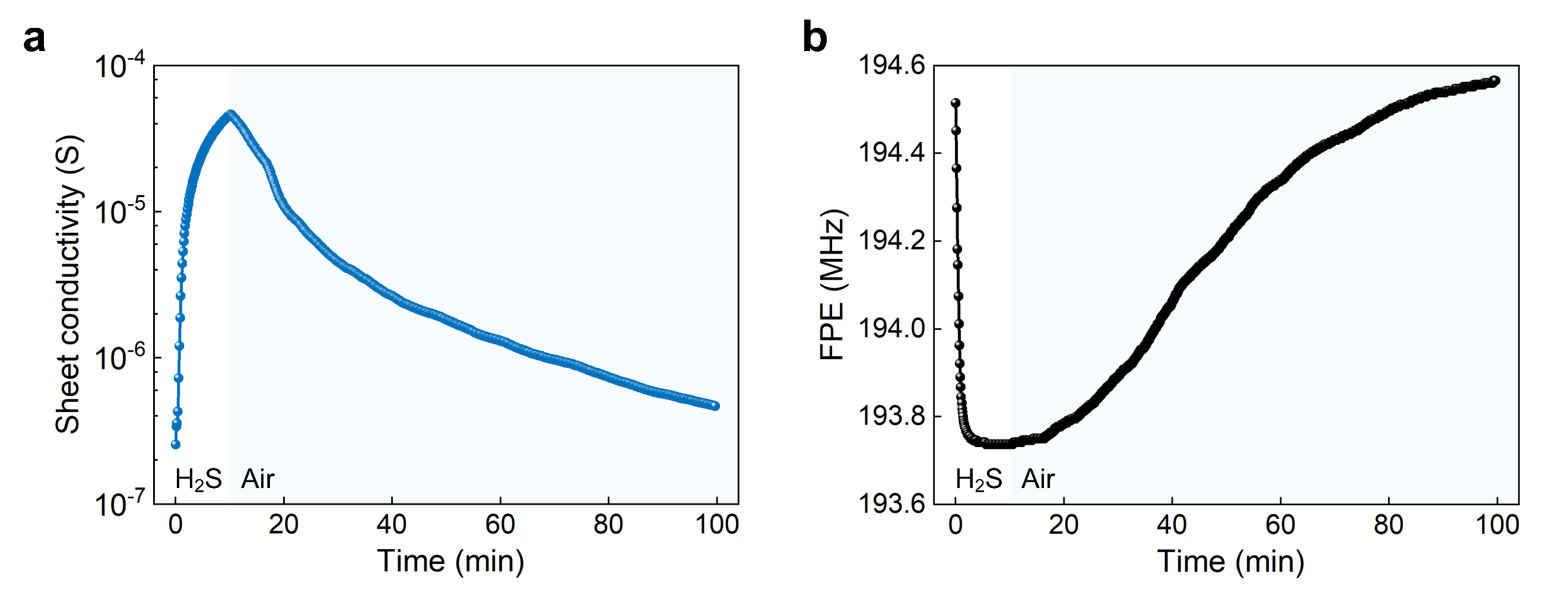


**Fig. S5.** The time domain sensing curves of the (a) film sheet conductivity and (b) frequency of the phase extremum, correspond to Fig. 7.
